# Enzymatic activity and immunoreactivity of Aca s 4, an alpha-amylase allergen from the storage mite *Acarus siro*

**DOI:** 10.1186/1471-2091-13-3

**Published:** 2012-01-31

**Authors:** Jana Pytelková, Martin Lepšík, Miloslav Šanda, Pavel Talacko, Lucie Marešová, Michael Mareš

**Affiliations:** 1 Institute of Organic Chemistry and Biochemistry, Academy of Sciences of the Czech Republic, 16610 Prague, Czech Republic

**Keywords:** Aca s 4, Acarus siro, α-amylases, group 4 mite allergens, storage mites

## Abstract

**Background:**

Enzymatic allergens of storage mites that contaminate stored food products are poorly characterized. We describe biochemical and immunological properties of the native alpha-amylase allergen Aca s 4 from *Acarus siro*, a medically important storage mite.

**Results:**

*A. siro *produced a high level of alpha-amylase activity attributed to Aca s 4. This enzyme was purified and identified by protein sequencing and LC-MS/MS analysis. Aca s 4 showed a distinct inhibition pattern and an unusual alpha-amylolytic activity with low sensitivity to activation by chloride ions. Homology modeling of Aca s 4 revealed a structural change in the chloride-binding site that may account for this activation pattern. Aca s 4 was recognized by IgE from house dust mite-sensitive patients, and potential epitopes for cross-reactivity with house dust mite group 4 allergens were found.

**Conclusions:**

We present the first protein-level characterization of a group 4 allergen from storage mites. Due to its high production and IgE reactivity, Aca s 4 is potentially relevant to allergic hypersensitivity.

## Background

Storage mites are global pests of stored food products of increasing medical and economical impact. In agricultural environments, they cause occupational allergy in farmers and grain handlers. Storage mites are also found in house dust from rural and urban dwellings and are important contributors to the allergen content, which expands their clinical significance. The storage mites belong to the Acaridae and Glycyphagidae families; our work focuses on *Acarus siro*, one of the most frequent and abundant species in central Europe.

More than two dozen groups of mite-derived allergens have been described in the WHO/IUIS Allergen Nomenclature database http://www.allergen.org. Allergens from house dust mites of *Dermatophagoides *spp. have been extensively studied; however, much less is known about allergens from storage mites (e.g. 7 records for *A. siro *allergens) (for review, see [1-3]). There is increasing evidence that mites contain epitopes that are species-specific as well as cross-reactive among species. The effect of a partial cross-reactivity between storage mites and house dust mites and co-sensitization by both groups further increases the medical impact of storage mites [4-9]. A detailed analysis of storage mite-derived allergens at the protein level will be necessary to better evaluate aspects of their sensitization specificity and biochemical activity, as well as to improve diagnosis and treatment.

Group 4 mite allergens are homologous proteins of the α-amylase class [10,11]. Group 4 allergens have been investigated in house dust mites such as *Dermatophagoides pteronyssinus, Euroglyphus maynei*, and *Blomia tropicalis*, and their sequences have been determined [12-14]. The biochemical properties of Der p 4 were analyzed in detail, including its interaction with major cereal flour allergens that act as α-amylase inhibitors [14,15]. The IgE-binding activity of group 4 allergens has been demonstrated for ~30% of allergic subjects in Western populations and China [12-14]; these allergens may also be the major contributor to the serum activity, as found in an Australian Aboriginal community [16]. In this work, we analyze native Aca s 4 from *A. siro*, the first α-amylase allergen to be isolated from storage mites. Specifically, we describe its biochemical and immunological properties. Furthermore, we provide insight into the 3D structure of Aca s 4 with the help of a novel homology model, the first 3D model of a group 4 allergen.

## Results and Discussion

### Quantification of α-amylase activity in *A. siro*

**Figure 1:**
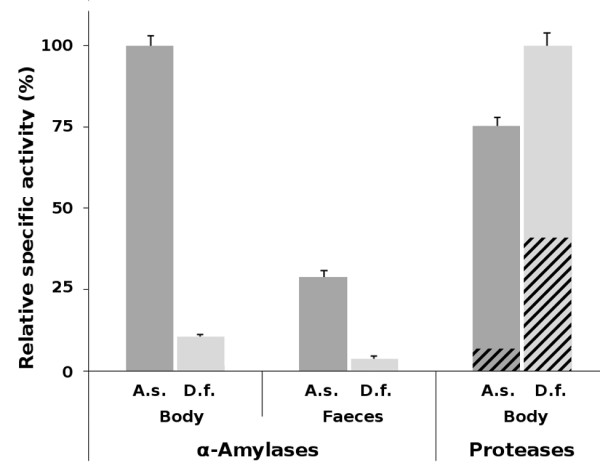
**Distribution of α-amylase and protease activities in the whole body extract and faecal extract of *A. siro *(A.s.) and *D. farinae *(D.f.)**. The α-amylase activities were assayed at the respective pH optima with RBB-starch as a substrate. The protease activities were assayed with azocasein as a substrate; the contribution of cysteine proteases (dashed) was determined as the part of protease activity inhibited by E-64. The specific activities (units per mg protein) are normalized to the maximum value measured for α-amylases and proteases, respectively; mean values ± SE are given.

A high α-amylase activity was demonstrated in the whole body extract from the storage mite *A. siro *using chromogenic starch as a substrate. Figure 1 shows that this activity was one order of magnitude higher than that measured for a model house dust mite *D. farinae *(specific activity 599.6 ± 18.0 and 64.1 ± 0.3 U.mg^-1 ^protein, respectively). In both species, a pronounced α-amylase activity was also detected in the faecal extract, suggesting that α-amylases are digestive enzymes secreted into the gut lumen and released in the mite faeces (Figure 1).

For comparison, the proteolytic activity was determined in the whole body extracts, which showed that *D. farinae *has a higher proteolytic activity than *A. siro *(specific activity 21.9 ± 0.9 and 16.5 ± 0.4 U.mg^-1 ^protein, respectively) and a higher content of cysteine proteases (Figure 1). We conclude that there is an important difference in the distribution of digestive enzymes in these model species of storage and house dust mites, which most likely reflects their feeding ecology. The high level of α-amylase activity in *A. siro *is in accordance with the feeding preferences of *A. siro*, a granivorous species evolutionarily adapted to utilization of a starch-rich diet [17].

### Isolation and proteomic identification of Aca s 4

**Figure 2:**
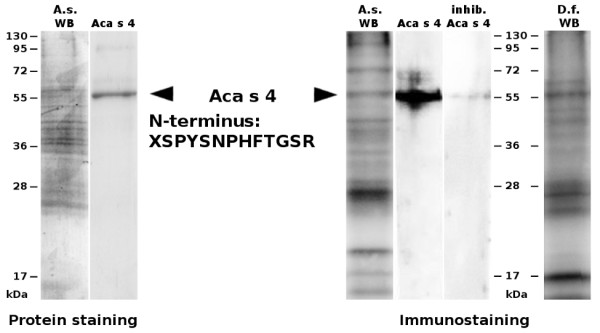
**Purification and IgE reactivity of Aca s 4**. The whole body extracts (20 μg) of *A. siro *(A.s WB) and *D. farinae *(D.f. WB) and the purified Aca s 4 (2.5 μg) were resolved by SDS-PAGE. Left-hand panel: A gel stained for protein with Coomassie blue. Right-hand panel: Western blot probed with pooled sera from mite-allergic patients sensitive to *Dermatophagoides *spp. and with anti-IgE antibodies and developed by chemiluminescence. For immunostaining inhibition (inhib.), the pooled sera were preincubated with purified Aca s 4. The arrows mark the position of Aca s 4 (~56 kDa) with the N-terminal sequence determined by Edman sequencing. Molecular mass standards are indicated.

**Figure 3:**
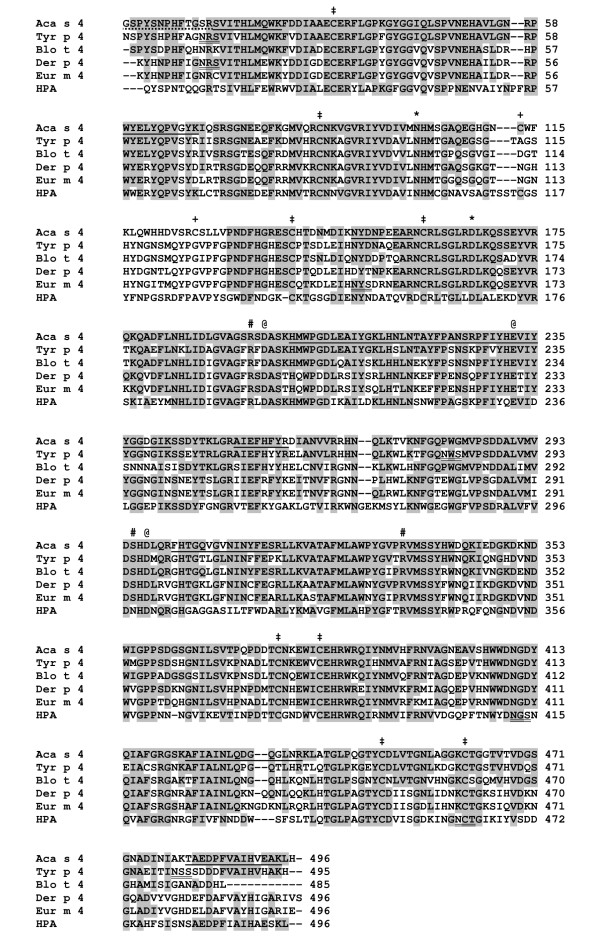
**Multiple sequence alignment of Aca s 4 with other α-amylases of mite origin and human α-amylase**. Aca s 4: *Acarus siro *(GenBank: ABL09312); Tyr p 4: *Tyrophagus putrescentiae *(GenBank: ABM53754); Blo t 4: *Blomia tropicalis *(GenBank: AAQ24543) [12]; Eur m 4: *Euroglyphus maynei *(GenBank: AAD38943) [13]; Der p 4: *Dermatophagoides pteronyssinus *(GenBank: AAD38942) [13]; HPA: human pancreatic α-amylase (GenBank: AAH07060). The sequence similarities to Aca s 4 are 74%, 70%, 64%, 66%, and 50%, respectively. Full-length sequences of mature proteins are aligned. Amino acids identical to those of Aca s 4 are shaded. In the Aca s 4 sequence, the N-terminal sequence determined by Edman sequencing (dotted underline) and fragments determined by LC-MS/MS analysis (solid underline) are indicated. Positions of catalytic residues (@) and residues binding the Cl^- ^(#) and the Ca^2+ ^(*) are marked. N-glycosylation signals are double underlined (Aca s 4 and Blo t 4 have no predicted N-glycosylation sites). Note the Asn295Ser mutation located in the Cl^- ^-binding site of mite α-amylases (compared to HPA and other animal α-amylases). Cys residues forming four conserved disulfide bridges (‡) and one specific disulfide (+) in the Aca s 4 molecule are marked.

α-Amylase was purified to homogeneity from the whole body extract of *A. siro *using an optimized procedure for affinity precipitation with glycogen. The typical yield was approximately 175 μg from 1 g of fresh weight of mites. The purified enzymatically active α-amylase migrated as a single band of 56 kDa on SDS-PAGE (Figure 2). We performed a two-pronged proteomic characterization of this protein: (i) the N-terminal amino acid sequence, XSPYSNPHFTGSR (X is an unidentified residue), was determined by Edman sequencing and (ii) the protein was subjected to enzymatic digestion followed by LC-MS/MS analysis. The data were searched against the UniProt protein database, which revealed identity with the cDNA-derived protein sequence of an *A. siro *α-amylase homolog denoted Aca s 4 (GenBank: ABL09312). The MS/MS peptide coverage of this sequence was ~31% (Figure 3). A theoretical mass calculated for the mature Aca s 4 (sequence starting at the native N-terminus) is 55956 Da, which is in good agreement with the experimental value obtained for the purified Aca s 4 (Figure 2).

### Biochemical functional characterization of Aca s 4

**Figure 4:**
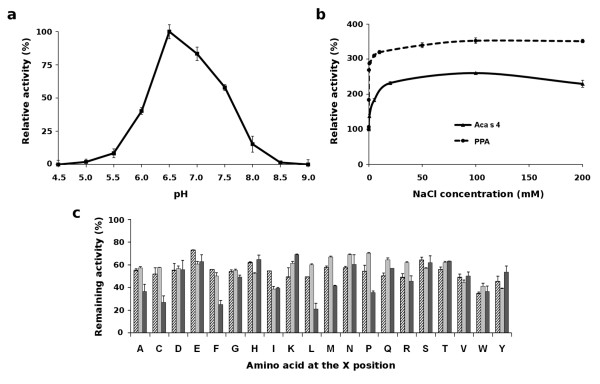
**Enzymatic properties of purified Aca s 4**. (a) pH profile determined with RBB-starch as a substrate. Mean values ± SE are normalized to the maximum value. (b) Activation effect of NaCl on amylolytic activity of Aca s 4 (solid line) and porcine pancreatic α-amylase (PPA) (dashed line) measured with the RBB-starch assay. Mean values ± SE are expressed as a percentage of activity relative to the control without NaCl (100%). (c) Inhibition profile of Aca s 4 determined by a library of PAMIs with the general structure Ac-XHWYYRCW-NH_2_; the X position contains one of the 20 naturally occurring amino acids as indicated. The inhibition sensitivity of the purified Aca s 4 (hatched) is compared to that of whole body extracts of *A. siro *(gray) and *D. farinae *(black). Mean values ± SE are expressed as the percentage of amylolytic activity (RBB-starch assay) relative to the uninhibited control (100%).

The purified Aca s 4 was characterized with regard to its substrate and inhibitor interactions. The pH profile (Figure 4a) shows that the enzyme functions in the slightly acidic to neutral range, with a maximum at pH ~6.5, which is in accordance with the pH optimum of α-amylase activity measured with the whole body extract of *A. siro *[17]. A similar pH optimum was also reported for the purified Der p 4 [14]. We tested the modulation of the Aca s 4 activity by chloride ions, which are general activators of animal α-amylases [18,19]. Figure 4b shows the activation of a typical chloride-dependent α-amylase from porcine pancreas and of Aca s 4 in the presence of NaCl. Chloride ions induced an increase in the activity of both Aca s 4 and its porcine homolog; however, Aca s 4 was activated to a lesser extent. Aca s 4 was inhibited by acarbose (IC_50 _~3.8 μM), a microbial oligosaccharide that is a general inhibitor of enzymes of the α-amylase class. This is in line with our previous finding that acarbose exerts an acaricidal activity against *A. siro *by inhibiting its digestive amylolytic activity [17]. Interestingly, Aca s 4 was insensitive to inhibition by two types of proteinaceous inhibitors of plant origin, namely wheat inhibitors WI-1 and WI-3 (tetrameric and monomeric form, respectively) and bean inhibitor αAI-1, which are potent inhibitors of various insect and mammalian α-amylases [20,21]. A pronounced inhibitory effect against Der p 4 has been reported for the tetrameric wheat inhibitor [15]. We applied a combinatorial library of synthetic PAMIs (Peptide α-Amylase Inhibitors) that was developed to analyze the inhibitory specificity of α-amylases [22]. Using this tool, we compared the inhibitory specificity of Aca s 4 and Der f 4 (an α-amylase of the house dust mite *D. farinae*, measured in the extract). Figure 4c shows that inhibition profiles of both enzymes follow the same general trend but also have distinct features. This result indicates that the active site regions of mite α-amylases share overall architecture but differ in some structural details. This analysis helps increase understanding of the different affinities of mite α-amylases to natural proteinaceous inhibitors such as the wheat α-amylase inhibitor [15].

### Three-dimensional model of Aca s 4

**Figure 5:**
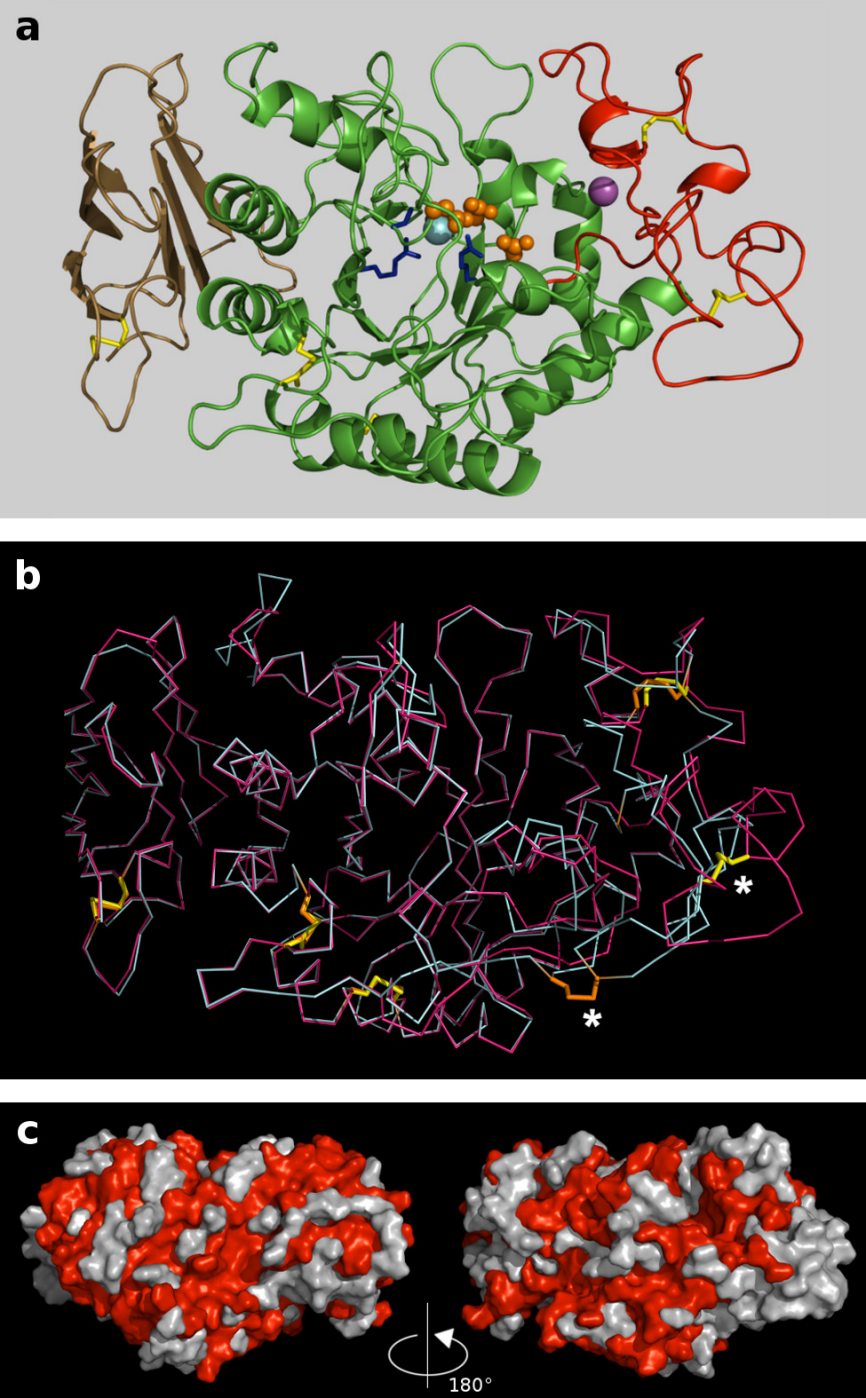
**Spatial model of Aca s 4 built by homology modeling and simulations**. (a) The Aca s 4 structure (in ribbon representation) is composed of the consensus α-amylase domains A (green), B (red), and C (brown). Three catalytic residues (D196, E232, D297) in the active site are highlighted (orange ball-and-stick). The calcium ion is depicted as a magenta sphere. The chloride binding site (blue sticks) is composed of the conserved binding residues R194 and R334 and the residue S295, which is specific for mite α-amylases; the chloride ion is shown as a light blue sphere. The disulfide bridges are represented by yellow sticks. (b) A superposition of Cα traces of Aca s 4 (magenta) with HPA (cyan; PDB: 2CPU) used as a template for Aca s 4 modeling. The disulfide bridges are represented by yellow (Aca s 4) and orange (HPA) sticks; non-conserved disulfides are marked by asterisks. (c) The surface model of Aca s 4; the right-hand view is in the same orientation as in (a). The molecule is colored red for residues that are identical for Aca s 4 and Der p 4.

A structural model of Aca s 4 was created by homology modeling (see Methods for details). The Aca s 4 structure shows an overall fold and secondary structure elements forming three consensus domains as in the insect and mammalian α-amylases (Figure 5a) [10,11]. The disulfide pattern is composed of four conserved disulfides and one additional disulfide (Cys113-Cys126) located in the B domain (Figure 3); Aca s 4 does not contain free-thiol cysteines, as demonstrated experimentally by a thiol labeling experiment (see Methods, data not shown). The catalytic center of Aca s 4 consists of three acidic residues and retains the characteristic α-amylase architecture (Figure 5a). We inspected the calcium-binding and chloride-binding sites, which are known to be important for active site function in the animal α-amylases. The structure of the calcium-binding site, which is necessary for the stabilization of the active site, is preserved in the Aca s 4 model. In contrast, the chloride-binding site of Aca s 4 was found to be modified in comparison with the structures of other animal α-amylases. The typical chloride-binding residues Arg194, Asn295 and Arg334 (Aca s 4 numbering) forming the "RNR signature" are not all conserved, and Asn295 is replaced by Ser in Aca s 4 (Figure 3). Furthermore, a comparison of the Aca s 4 sequence with those of its homologs available in the sequence database showed that this substitution (resulting in RSR signature of the chloride-binding site) is specific for α-amylases of mite origin (Figure 3). The chloride ion serves as an allosteric activator of catalysis of α-amylases [18,19]. The lower sensitivity of Aca s 4 to chloride activation (Figure 4a) is likely due to the substitution of Asn with Ser in the chloride-binding site. This is supported by a study performed on human pancreatic α-amylase in which the chloride-binding residues were mutated [18]; the substitution at position 295 resulted in a defect in catalytic efficiency and chloride binding that resembles the behavior of Aca s 4. Recently, we have described another evolutionarily acquired mutation (Arg-to-Gln334) in the chloride-binding site that leads to the chloride-independence of alkaline α-amylases of lepidopteran insects [21].

### Immunoreactivity of Aca s 4

The IgE reactivity of Aca s 4 was tested by immunoblotting using pooled sera from patients allergic to house dust mites (see Methods). Figure 2 shows binding of IgE to the purified Aca s 4; the staining was specific, as it was inhibited when using pooled sera preincubated with Aca s 4. The staining pattern of the whole body extract of *A. siro *demonstrated that Aca s 4 of ~56 kDa is a significant IgE-reactive component although it is a protein of low abundance in the *A. siro *extract. Aca s 4 is likely recognized by serum IgE due to (i) a cross-reactivity with homologous α-amylase allergens from house dust mites (see a ~56 kDa band immunostained in the whole body extract of *D. farinae *in Figure 2) and/or (ii) a reactivity of anti-Aca s 4 IgE induced by exposure to *A. siro *(most likely during co-sensitization with house dust mites). To gain insight into the structural basis of a possible cross-reactivity, we compared Aca s 4 with Der p 4, an α-amylase allergen from *D. pteronyssinus *with known sequence (GenBank: AAD38942; [13]). Both sequences display a 66% amino acid identity. The conserved sequence regions are shown on the surface model of Aca s 4 (Figure 5b); they form a dense net of clusters representing the potential common IgE-binding epitopes.

### Conclusions

Our work provides the first comprehensive protein-level analysis of the α-amylase allergen Aca s 4 from *A. siro*. The results give new insights into the biochemistry of the group 4 allergens of mites and suggest that the interaction of Aca s 4 with patients' IgE may be relevant to allergic hypersensitivity to mites.

## Methods

### Materials

Enzyme inhibitors: Acarbose was obtained from Bayer (Berlin, Germany); WI-1, WI-3 and E-64 from Sigma (St. Louis, MO); and Pefabloc from Roche (Indianapolis, IN). αAI-1 was isolated according to [20]; the development and synthesis of PAMIs is described in [22]. The substrates and enzymes: Remazol Brilliant Blue dyed starch (RBB-Starch) and azocasein were purchased from Fluka (Buchs, Switzerland), porcine pancreatic α-amylase (PPA) from Sigma. The *A. siro *and *D. farinae *originated from laboratory cultures that were maintained and mass-reared as previously described [17,23]. Live mites were collected from the stock culture and washed; the faeces were separated from the spent growth medium by sieving [23]; these materials were stored at -80°C. The pooled serum was prepared from serum samples collected from 33 subjects from the Czech Republic with allergies to house dust mites. The patient sera had specific IgE levels against *D. farinae *and/or *D. pteronyssinus *above 0.72 kU/l (scores of class 2 to 6); the titer was determined using an IgE-capture immunoenzymatic ALLERGEN System (Radim Diagnostics, Pomezia, Italy).

### Protein extracts

The biological samples (mite bodies or faeces) were homogenized (50 mg fresh weight per mL) on ice in 50 mM MES, pH 6.0, containing 5 mM CaCl_2_, 0.1 M NaCl, 25% glycerol, 0.02% NaN_3_, and protease inhibitors (10 μM E-64 and 1 mM Pefabloc). The inhibitors were not included in the extracts used for proteolytic activity measurements. The homogenate was centrifuged (10000 g, 10 min, 4°C), and the supernatant was filtered with a Micropure-0.22 Separator (Millipore, Bedford, MA); the final extracts were stored at -80°C. The protein content was quantified by bicinchoninic acid protein assay (Pierce, Rockford, IL).

### Isolation of Aca s 4

The purification procedure was based on a previously described method [21]. The whole body extract of *A. siro *was prepared in 50 mM Na acetate, pH 5.0, containing 5 mM CaCl_2_, 10% glycerol and protease inhibitors (10 μM E-64 and 1 mM Pefabloc). Ethanol was added to a final concentration of 40%, keeping the samples on ice, and the mixture was centrifuged (10000 g, 10 min, 4°C). The supernatant was treated with 0.2% glycogen for 5 min on ice, and the α-amylase-glycogen complex was collected by centrifugation (10000 g, 10 min, 4°C) and washed with the extraction buffer containing 40% ethanol. The final sediment was incubated with rotation (2 h, 26°C) in 50 mM MES, pH 6.0, containing 5 mM CaCl_2 _and 10% glycerol and dialyzed against the same buffer. The purity of the isolated Aca s 4 was confirmed by Laemmli SDS-PAGE, and its concentration was determined by bicinchoninic acid protein assay.

### Proteomic methods

Mass spectrometric characterization of Aca s 4 was performed by LC-MS/MS analysis of the tryptic digest. The LC-MS/MS analysis was performed on a LTQ Orbitrap XL hybrid mass spectrometer (Thermo Scientific, Waltham, MA) coupled to a Rheos 2000 2D capillary HPLC system (Flux instruments, Basel, Switzerland). The first dimension column was a monolithic PS-DVB (200 μm × 10 mm, Dionex, Sunnyvale, CA), and the second dimension column was a C18 PepMap 100 (75 μm × 150 mm × 3 μm, Dionex) with gradient elution in a 0.1% formic acid/acetonitrile system. The LC-MS/MS data were processed with Sequest and Bioworks software (Thermo Scientific) and searched against the UniProt protein database http://www.uniprot.org. N-terminal Edman sequencing of Aca s 4 was performed by using a Procise 494 cLC protein sequencer (Applied Biosystems, Carlsbad, CA). The amino acid sequences were searched by BLAST http://blast.ncbi.nlm.nih.gov and aligned by ClustalW http://www.ebi.ac.uk/Tools/msa/clustalw2. The labeling experiment for the detection of free-thiol cysteines in Aca s 4 was performed with 5-iodoacetamidofluorescein (Molecular Probes, Eugene, OR) under denaturing conditions followed by SDS-PAGE visualization as previously described [24].

### Enzyme activity and inhibition assays

α-Amylase activity was assayed with the chromogenic substrate RBB-starch. An enzyme aliquot was incubated (20 min, 26°C) with 0.3% RBB-starch in 0.1 M Britton-Robinson buffer at the pH optimum of the enzyme (6.5 for Aca s 4 and *A. siro *extract, 7.0 for *D. farinae *extract, 6.9 for PPA) or at pH 4.5-9.0 (pH profiling). The reaction was stopped with 0.2 M NaOH, the mixture was centrifuged (10000 g, 10 min), and the absorbance at 620 nm of the supernatant was measured against a control sample (incubated in the absence of enzyme/extract). Typically, 0.35 U of α-amylase activity at the pH optimum was used in the assay (1 U produces A_620 nm _= 1). For the activity assay in the presence of α-amylase inhibitors, an enzyme aliquot was preincubated (20 min, 26°C) in the assay buffer with the following inhibitor concentrations: 10 μM WI-1, WI-3, or αAI-1; 0.1-10 μM acarbose; 10 μM PAMI for *D. farinae *extract and 50 μM PAMI for Aca s 4 and *A. siro *extract. For the activity assay in the presence of NaCl, enzyme samples were dialyzed against water. The measurements were performed in triplicate. Proteolytic activity was assayed with the chromogenic substrate azocasein at pH 6.0 essentially as described [25]. The inhibition of cysteine proteases in the extract was performed by preincubation (10 min, 26°C) with 10 μM E-64.

### Homology modeling and molecular simulations

A 3D model of Aca s 4 was created with the SwissModel server [26] using the Aca s 4 sequence GenBank: ABL09312 and the X-ray structure of human pancreatic α-amylase (HPA) (PDB: 2CPU) as a template. The model did not include the terminal residues 1-4 and 496 due to the lack of homology with the human enzyme (residue numbering is according to the mature Aca s 4 sequence). The Ca^2+ ^and Cl^- ^ions were inserted manually according to the 2CPU structure. Four conserved disulfide bridges (linking positions 31-87, 140-159, 376-382,449-461) were constructed automatically by SwissModel, while an additional disulfide connecting Cys113 and Cys126 was modeled using the following simulation protocol: (i) minimization of the added hydrogens, (ii) minimization and molecular dynamics of the segment 104-133: 50 ps at 10 K with a restraint (5 to 500 kcal.mol^-1^.Å^-1^) on the disulfide S-S distance, (iii) molecular dynamics of the segments extending to 43-167: 50 ps at 300 K, and (iv) minimization of the segment spanning residues 43-167. The non-conserved disulfides Cys113-Cys126 of Aca s 4 and Cys70-Cys115 of HPA are associated with conformational differences in domain B. The final Aca s 4 structure was validated using Molprobity [27] and deposited in the Protein Model Data Bank http://mi.caspur.it/PMDB/ under the accession code PM0077555. Structure figures were prepared with PyMOL (DeLano Scientific LLC, San Carlos, CA).

### Electrophoretic and immunological methods

The proteins were separated by reducing Laemmli SDS-PAGE (15% gel), then stained with Coomassie blue or, for immunostaining transferred to a PVDF membrane by electroblotting. Immunoblots were developed using pooled patients' sera (diluted 1:1000 in 10 mM Tris-HCl, pH 7.4, containing 150 mM NaCl and 0.05% Tween 20), anti-human IgE antibody conjugated with horseradish peroxidase (Sigma) (1:25000), and SuperSignal West Pico chemiluminescent substrate (Pierce). Protein molecular mass standards (PageRuler Plus Prestained Protein Ladder, 10-250 kDa, Fermentas, Burlington, Canada) were not immunostained under these conditions. For the inhibition experiment, the pooled sera were preincubated with purified Aca s 4 (1 μg/10 μl serum). The blots were visualized with a LAS-4000 luminescent image analyzer (Fujifilm, Valhalla, NY).

## Abbreviations

Aca s 4: a group 4 allergen from *Acarus siro*; Der p 4 and Der f 4: a group 4 allergen from *Dermatophagoides pteronyssinus *and *D. farinae*, respectively; PPA and HPA: porcine and human pancreatic α-amylase, respectively; RBB-Starch: Remazol Brilliant Blue dyed starch; PAMI: peptide α-amylase inhibitor; WI-1 and WI-3: wheat α-amylase inhibitors 1 and 3, respectively; αAI-1: bean α-amylase inhibitor 1; E-64: *N*-[*N*-(L-3-*trans*-carboxyirane-2-carbonyl)-L-leucyl]-agmatine.

## Authors' contributions

JP carried out the biochemical and immunological studies. ML carried out the molecular modeling. MS performed the proteomic analysis. PT participated in the immunological analysis. LM participated in the enzyme inhibition analysis. MM designed the study and wrote the manuscript. All authors read and approved the final manuscript.
